# The eyes have it: pupillary assessment as a measure of sleep and circadian health

**DOI:** 10.1093/sleep/zsae285

**Published:** 2024-12-19

**Authors:** Sabra M Abbott

**Affiliations:** Department of Neurology, Northwestern University Feinberg School of Medicine, Chicago, IL, USA

In our 24-hour environment, daily light exposure from the rising and setting of the sun has long been recognized as a key time cue or *zeitgeber* in the regulation of circadian rhythms. Artificial light exposure at inappropriate circadian times can impair this entrainment, while exposure to a solely natural light environment has been demonstrated to re-entrain individuals with a wide range of baseline sleep–wake schedules [1].

The retina contains multiple photoreceptors; however, the primary photoreceptors responsible for transmitting light information to the suprachiasmatic nucleus (SCN) to influence circadian timing are intrinsically photosensitive retinal ganglion cells (ipRGCs), containing the photopigment melanopsin [2]. In addition to sending projections to the SCN, the ipRGCs also project to the brainstem, where they mediate pupillary constriction [3]. More specifically, the post-illumination pupillary light response (PIPR) is a measure of sustained pupil constriction following removal of a light stimulus which correlates with ipRGC function [4]. As a result, pupillary light measures can be utilized as a clinical marker for the overall function of the neuronal system responsible for circadian light perception.

Previous studies have evaluated the role of ipRGC function in disorders that are known to be associated with impaired light perception and circadian dysfunction. For example, seasonal affective disorder (SAD) is a condition in which depressive symptoms become worse during times of shorter day length. Studies have demonstrated an attenuated PIPR in individuals with SAD suggesting that a decreased ability to respond to light may contribute to this disorder’s underlying pathology [5]. In the circadian rhythm disorder non-24-hour sleep–wake rhythm disorder (N24SWD) individuals typically follow a daily rhythm that is slightly longer than 24 hours, as evidenced by patterns of falling asleep and waking up slightly later each day. While first described in blind individuals, presumably due to a lack of photic input to the SCN, a subset of individuals with normal image forming vision exist, frequently referred to as “sighted N24SWD.” Pupillometry studies in those individuals have also demonstrated an attenuated PIPR, suggesting that while they have retained image forming vision, the circadian light input to the SCN is impaired. Similar findings have also been found with individuals with delayed sleep–wake phase disorder who have extremely late bedtimes [6].

The recent study by Chan et al. moves these observations into a larger clinical population, looking at the PIPR in relation to both objective and subjective measures of circadian amplitude [7]. It has long been recognized that aging is associated with a decrease in circadian amplitude across multiple measures, which may in turn relate to the decreased sleep efficiency seen in older adults [8]. Given the high prevalence of ocular disease in the aging population (e.g. glaucoma, macular degeneration), previously studies have focused on whether these broad ocular pathologies have been associated with impaired sleep and decreased circadian amplitude [9–11]. However, those same associations have not previously been evaluated in healthy older individuals without known ocular disease.

Chan et al. evaluated a healthy population of older adults, with no evidence of cognitive impairment, and no known ocular pathology. In this population, a correlation was demonstrated between the presence of an attenuated PIPR and a decrease in the amplitude of urinary 6-sulfatoxymelatonin production and rest-activity behaviors as measured by actigraphy. These associations remained present after adjusting for age, day length, timing of testing, and prior light exposure. While studies are still needed to determine whether enhanced light exposure results in improved circadian amplitude in otherwise healthy individuals with an attenuated PIPR, this suggests a potentially intervenable target to improve sleep and circadian health in the aging individual.

There are some practical examples of how this may be effective when looking at populations with neurodegenerative disease. For example, there is evidence that individuals with Parkinson disease have retinal degeneration and loss of ipRGCs [12]. Light-based interventions with twice daily bright light exposure have improved not only sleep-related symptoms in Parkinson disease, but have also demonstrated improvements in motor dysfunction [13]. Similarly, individuals with Alzheimer disease also exhibit evidence of retinal neurodegeneration and loss of ipRGCs, with associated fragmentation and decreased amplitude of rest-activity patterns [14]. Studies have demonstrated that bright light therapy can be beneficial for addressing sleep disturbances, as well as affective symptoms, particularly in patients with mild to moderate Alzheimer disease [15].

Based on these findings, one can envision the sleep and circadian medicine clinic of the future implementing routine pupillary assessments to determine which individuals may benefit most from enhanced light interventions as a strategy to improve overall sleep and circadian health.
